# Cardiac surgery outcome during the COVID-19 pandemic: a retrospective review of the early experience in nine UK centres

**DOI:** 10.1186/s13019-021-01424-y

**Published:** 2021-03-22

**Authors:** Julie Sanders, Enoch Akowuah, Jackie Cooper, Bilal H. Kirmani, Mazyar Kanani, Metesh Acharya, Reuben Jeganathan, George Krasopoulos, Dumbor Ngaage, Indu Deglurkar, Patrick Yiu, Simon Kendall, Aung Ye Oo

**Affiliations:** 1 St Bartholomew’s Hospital, Barts Health NHS Trust, London, EC1A 7DN UK; 2 William Harvey Research Institute, Queen Mary University of London, London, UK; 3 Department of Cardiothoracic Surgery, James Cook University Hospital, South Tees NHS Foundation Trust, Middlesbrough, UK; 4 Department of Cardiothoracic Surgery, Liverpool Heart and Chest Hospital, Liverpool, UK; 5 Department of Cardiothoracic Surgery, Glenfield Hospital, University Hospitals Leicester NHS Foundation Trust, Leicester, UK; 6 Department of Cardiothoracic Surgery, Royal Victoria Hospital, Belfast, Northern Ireland, UK; 7 Department of Cardiothoracic Surgery, Oxford University Hospitals NHS Foundation Trust, Oxford, UK; 8 Department of Cardiothoracic Surgery, Castle Hill Hospital, Hull, UK; 9 Department of Cardiothoracic Surgery, University Hospital of Wales, Cardiff, Wales, UK; 10 Heart and Lung Centre, New Cross Hospital, Wolverhampton, UK

**Keywords:** Covid-19, Cardiac surgery, Mortality, Outcomes

## Abstract

**Background:**

Early studies conclude patients with Covid-19 have a high risk of death, but no studies specifically explore cardiac surgery outcome. We investigate UK cardiac surgery outcomes during the early phase of the Covid-19 pandemic.

**Methods:**

This retrospective observational study included all adult patients undergoing cardiac surgery between 1st March and 30th April 2020 in nine UK centres. Data was obtained and linked locally from the National Institute for Cardiovascular Outcomes Research Adult Cardiac Surgery database, the Intensive Care National Audit and Research Centre database and local electronic systems. The anonymised datasets were analysed by the lead centre. Statistical analysis included descriptive statistics, propensity score matching (PSM), conditional logistic regression and hierarchical quantile regression.

**Results:**

Of 755 included individuals, 53 (7.0%) had Covid-19. Comparing those with and without Covid-19, those with Covid-19 had increased mortality (24.5% v 3.5%, *p* < 0.0001) and longer post-operative stay (11 days v 6 days, *p* = 0.001), both of which remained significant after PSM. Patients with a pre-operative Covid-19 diagnosis recovered in a similar way to non-Covid-19 patients. However, those with a post-operative Covid-19 diagnosis remained in hospital for an additional 5 days (12 days v 7 days, *p* = 0.024) and had a considerably higher mortality rate compared to those with a pre-operative diagnosis (37.1% v 0.0%, *p* = 0.005).

**Conclusions:**

To mitigate against the risks of Covid-19, particularly the post-operative burden, robust and effective pre-surgery diagnosis protocols alongside effective strategies to maintain a Covid-19 free environment are needed. Dedicated cardiac surgery hubs could be valuable in achieving safe and continual delivery of cardiac surgery.

## Background

On 30 January 2020, the World Health Organisation (WHO) declared the outbreak of Covid-19 (SARS-CoV-2) a Public Health Emergency of International Concern. As of 23rd November 2020, there were over 58 million cases and almost 1.4 million deaths worldwide, including 371,419 deaths in Europe where the United Kingdom (UK), Italy, France, Spain and Russia have reported the highest mortality [1]. In people with cardiovascular disease, the prognosis of Covid-19 is particularly poor [2]. In the UK, ischaemic heart disease was the most common main pre-existing medical condition reported in 14% of all deaths involving Covid-19 [3].

Cardiothoracic practice may not be in the frontline of the Coronavirus response but the service must respond to the demands of the pandemic, and in doing so has been considerably affected. A worldwide survey spanning 60 cardiac surgery centres has identified that cardiac surgery reduced by 50–75% during the pandemic, with a > 50% reduction in dedicated cardiac theatre rooms and ICU beds [4]. In England, all National Health Service (NHS) hospitals were told by NHS England’s Chief Executive to suspend all elective surgery from 15th April for at least 3 months [5], although many cardiothoracic centres, especially those in London, did so from mid-March. However, the best local solution to continue the proper management of urgent and emergency, patients while protecting resources for the response to Coronavirus, was needed [6].

During a pandemic the primary focus is retained on the pandemic itself and critical care and so there is a paucity of information relating to surgical services and patient outcomes. Of the available current evidence relating to Covid-19, most are small studies (*n* < 35) concentrated on outcomes of Covid-19 positive patients following various surgical procedures [7], including thoracic [7, 8] and orthopaedic [9] surgery. These early studies conclude that surgery in those with Covid-19 have a high risk of death [7, 8] and that surgery may accelerate disease progression in those incubating Covid-19 [7]. More recently the COVIDSurg collaborative has reported on 1128 surgical patients (various clinical indications and surgery types) with a Covid-19 diagnosis (pre- or post-surgery) across 235 hospitals in 24 countries with a Covid-19 diagnosis, identifying a high 30 day mortality (23.8%) [10]. However, no studies or reports relating specifically to cardiac surgery outcomes during any pandemic have been identified.

The aim of this study is to describe and explore cardiac surgery outcomes during the first 2 months of the Covid-19 pandemic in the UK.

## Methods

### Patient population

All cardiac surgery centres in the UK were invited to participate in this study. The WHO defined Covid-19 as a pandemic on 11th March 2020. However, clinical services in the UK had already started planning and responding to the crisis, had already treated a number of patients with Covid-19 and almost all elective cardiac surgery had been suspended. Thus, to capture the start of the clinical cardiac surgery response to the pandemic, all patients undergoing any form of adult (> 18 years old) cardiac surgery between 1st March 2020 and 30th April 2020 were included.

### Data sources

Data from the National Adult Cardiac Surgery Audit (NACSA) database was linked to Intensive Care National Audit and Research Centre (ICNARC) data and local existing databases (for Covid-19 related data) at each participating centre. The linked dataset was anonymised locally and submitted to St Bartholomew’s Hospital.

### Covid-19

A standardised national Covid-19 swabbing protocol was not in place, and within centres, diagnosis protocols were not uniform. Most relied on one negative swab result anytime between one to 7 days prior to surgery. None undertook CT scanning at that time. Thus, for the purposes of this study a diagnosis of Covid-19 was defined as having a positive swab test or, where no test was performed, suspected clinically at any time pre- or post-surgery during the index hospital admission. This was reflective of current practice at this time.

### Data transfer and data storage

The anonymised dataset from each centre was transferred via an AWS (Amazon Web Service) S3 bucket. Submission to S3 via the S3 console is secure, using HTTPS/SSL protocols. Each centre was allocated a user login, restricted only to the uploading of data files to the bucket. Access to download and archive of the submitted files into a designated archive sub-folder was restricted to the St Bartholomew’s research team only.

### Data analysis and statistical methods

**Fig. 1:**
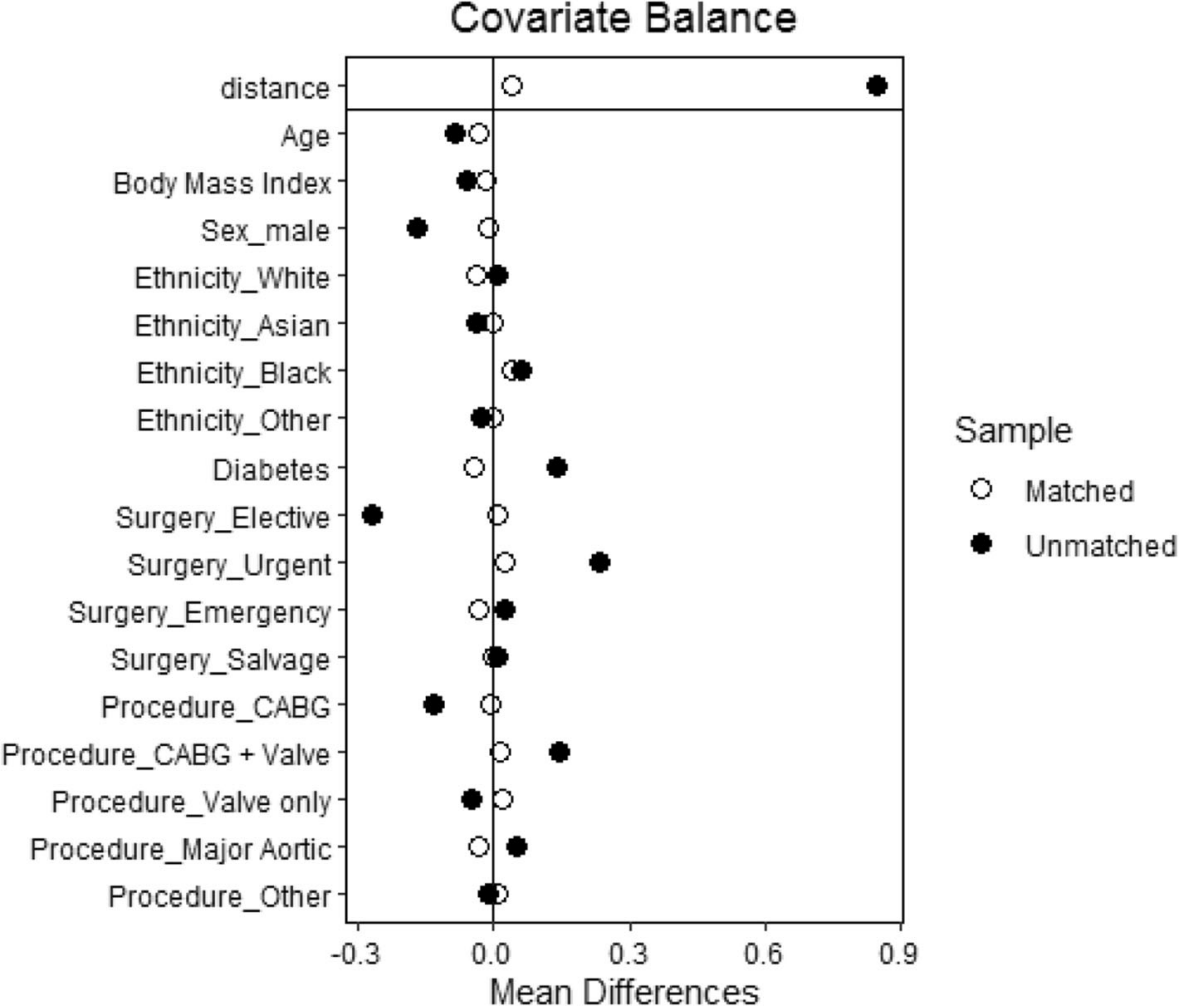
Covariate balance measured by standardised mean difference between patients without and with Covid-19 before and after propensity score matching

Results are presented as mean (SD) or median [IQR] for continuous variables and groups are compared using Mann-Whitney U test. Categorical data are presented as N(%) and compared using chi-squared or Fisher’s exact tests. Propensity score matching was used to assess the impact of Covid-19 on mortality and length of stay after accounting for potential covariates. We used optimal matching with a caliper width of 0.2 standard deviations. Three controls were matched to each Covid-19 case using age, centre, sex, ethnicity, diabetes, BMI, procedure type and urgency, resulting in improved balance between the groups as measured by the standardised mean differences (Fig. 1). Missing values were imputed prior to matching using a random forest algorithm [11]. Matched analysis was undertaken using conditional logistic regression and hierarchical quantile regression. Sensitivity analysis conducted using Firth logistic regression and quantile regression in a complete case analysis confirmed the results.

## Results

In total, nine centres (28% of all UK centres) from England, Wales and Northern Ireland participated in this study. Analysis is on 755 individuals undergoing cardiac surgery procedures between 1st March and 30th April 2020. Fifty-three (7.0%) patients were diagnosed with Covid-19 either pre- (*n* = 17) or post-operatively (*n* = 35), with the timing of diagnosis missing in *n* = 1. During the same period in 2019, 1582 cardiac surgery procedures were undertaken representing a 52% reduction in surgical activity.

### Patient characteristics

Table 1 details the baseline characteristics of the study cohort. Overall, patients were predominantly male (76.4%), of white background (87.1%) with a median age of 66 [57–72] years. Most presented for urgent (require surgery on this admission) (52.3%) or emergency (7.0%) first-time cardiac surgery (95.3%), staying in ICU for a median of 2 days and in hospital after the surgery for 6 days.

**Table 1:** Baseline characteristics. All definitions as per NACSA and ICNARC datasets

Variable	All Frequency (%)/mean ± SD or Median [IQR] (*n* = 755)	Covid-19 negative Frequency (%)/mean ± SD or Median[IQR] (*n* = 702)	Covid-19 positive Frequency (%)/mean ± SD or Median[IQR] (*n* = 53)	p (Covid-19 positive vs Covid-19 negative)
**Demographics**				
Age (years/median [IQR])	66 [57–72]	66 [57–72]	65 [57–72]	0.822
Female gender	178 (23.6)	174 (24.8)	4 (7.6)	0.004
Ethnicity				
White	392 (87.1)	362 (86.8)	30 (90.9)	0.046
Asian	26. (5.8)	26 (6.2)	0 (0.0)	
Black	12 (2.7)	9. (2.2)	3 (9.1)	
Other	20. (4.4)	20 (4.8)	0 (0.0)	
**Medical history**				
Previous MI (yes)	287 (51.9)	266 (52.6)	21 (44.7)	0.301
Previous PCI (yes)	105 (14.1)	95 (13.8)	10 (18.9)	0.302
Previous cardiac surgery (yes)	35 (4.7)	31 (4.4)	4 (7.6)	0.301
Renal function/dialysis	13 (1.9)	11 (1.7)	2 (4.3)	0.215
History of pulmonary disease	111 (16.1)	101 (15.6)	10 (25.0)	0.122
History of neurological disease	74 (9.9)	72 (10.4)	2 (3.9)	0.153
History of neurological dysfunction	18 (2.6)	17 (2.6)	1 (2.1)	1.00
**Symptoms**				
NHYA class				
I/II	406 (60.3)	380 (60.8)	26 (54.2)	0.365
III/IV	267 (39.7)	245 (39.2)	22 (45.8)	
CCSC classification				
0/I/II	480 (63.8)	445 (63.7)	35 (66.0)	0.729
IIII/IV	272 (36.2)	254 (36.3)	18 (34.0)	
**Cardiac risk factors**				
Smoking				
Never	326 (44.0)	302 (43.9)	24 (45.3)	0.942
Ex	309 (41.7)	288 (41.9)	21 (39.6)	
Current	106 (14.3)	98 (14.2)	8 (15.1)	
Hypertension	521 (69.8)	481 (69.3)	40 (75.5)	0.346
Diabetes	197 (26.3)	176 (25.3)	21 (39.6)	0.023
BMI (kg/m^2^/median [IQR])	28.1 [25.0–31.6]	28.1 [25.0–31.6]	27.1 [24.7–31.8]	0.375
**Examination and investigation**				
LVEF				
Good	514 (69.3)	480 (69.7)	34 (64.2)	0.496
Moderate/fair	178 (24.0)	163 (23.7)	15 (28.3)	
Poor	43 (5.8)	40 (5.8)	3 (5.7)	
Very poor	7 (0.9)	6 (0.9)	1 (1.9)	
Number of diseased vessels				
0	218 (32.4)	206 (32.9)	12 (26.7)	0.126
1	66 (9.8)	62 (9.9)	4 (8.9)	
2	95 (14.1)	83 (13.2)	12 (26.7)	
3	293 (43.6)	276 (44.0)	17 (37.8)	
Left main stem disease	144 (21.9)	136 (22.2)	8 (17.8)	0.578
Creatinine at time of surgery (umol/L)	87 [74–102]	86 [73–101.5]	96 [82.5–115]	< 0.0001
Pre-operative heart rhythm				
Sinus rhythm	643 (86.1)	598 (86.2)	45 (84.9)	0.888
Atrial fibrillation/flutter	80 (10.7)	73 (10.5)	7 (13.2)	
Complete Heart Block/pacing	17 (2.3)	16 (2.3)	1 (1.9)	
Ventricular tachycardiac/fibrillation	2 (0.3)	2 (0.3)	0 (0.0)	
Other abnormal	5 (0.7)	5 (0.7)	0 (0.0)	
**Pre-operative risk assessment**				
EuroSCORE (median [IQR])	2.3 [1.4–5.2]	2.3 [1.4–4.9]	3.9 [1.6–6.5]	0.015
**Intra-operative details**				
Operative priority				
Elective	298 (39.8)	291 (41.8)	7 (13.7)	< 0.001
Urgent	391 (52.3)	353 (50.7)	38 (74.5)	
Emergency	52 (7.0)	47 (6.7)	5 (9.8)	
Salvage	7 (0.9)	6 (0.9)	1 (2.0)	
Operation performed				
CABG	318 (46.0)	306 (47.1)	12 (29.3)	0.004
CABG+valve (any)	68 (9.8)	57 (8.8)	11 (26.8)	
Valve only (any)	181 (26.2)	173 (26.6)	8 (19.5)	
Major aortic	103 (14.9)	94 (14.5)	9 (22.0)	
Other	21 (3.0)	20 (3.1)	1 (2.4)	
Cardiopulmonary bypass used (Yes)	677 (95.1)	625 (94.8)	52 (98.1)	0.506
Cardiopulmonary bypass time (minutes)	104 [77–138]	102 [75–137]	116 [87–153]	0.020
Aortic cross clamp time (minutes)	72 [52–101]	70 [51–100]	84 [62–119]	0.024
**Outcome**				
Length of ventilation (days)	2 [1–2]	1.5 [1–2]	2 [1–2.5]	0.666
Length of ICU stay (days)	2 [1–4]	2 [1–4]	2 [1–3.5]	0.767
Return to theatre	34 (4.9)	33 (5.1)	1 (2.1)	0.723
Deep sternal wound infection	1 (0.2)	1 (0.2)	0 (0.0)	1.000
New neurological dysfunction	20 (3.0)	19 (3.0)	1 (2.1)	1.000
New haemofiltration/ dialysis	25 (3.7)	23 (3.6)	2 (4.3)	0.687
Post-operative length of stay (days)	6 [5–10]	6 [5–9]	11 [6–15]	0.0001
In-hospital mortality	37 (5.0)	24 (3.5)	13 (24.5)	< 0.0001

### Comparison of patients with and without Covid-19

Comparing those with (*n* = 53) and without (*n* = 702) Covid-19 found those with Covid-19 were more likely to be male (96.0% v 75.2%, *p* = 0.004), of white background (90.9% v 86.8%, *p* = 0.046), diabetic (39.6% v 25.3%, *p* = 0.023), have higher pre-operative serum creatinine levels (96umol/L [82.5umol/L v 115umol/L], v 86umol/L [73umol/L − 101.5umol/L], *p* < 0.001) and greater calculated EuroSCORE II (3.91 [1.7–6.5] v 2.26 [1.4–4.9], *p* < 0.015) (Table 1). Those with Covid-19 were also more likely to undergo urgent (74.5% v 50.7%, *p* < 0.001) and more complex surgery (for example, combined CABG and valve surgery 26.8% v 8.8%; major aortic surgery 22.0% v 14.5%, *p* = 0.004) with longer cardiopulmonary bypass (116 [87–153] v 102 [75–127], *p* = 0.02) and aortic cross clamp times (84 [62–119] v 70 [51–100], *p* = 0.024) as a result. While there were no differences in ICU length of stay (LOS) or in-hospital post-operative morbidity, those diagnosed with Covid-19 remained in hospital a median of 5 days longer after surgery than those without Covid-19 (11 [6–15] v 6 [5–9], *p* = 0.001), and had an overall median hospital stay of 6 days more than those without it (15 [12–21 v 9 [7–15], *p* < 0.0001). Furthermore, the in-hospital mortality rate was seven times higher in those with (24.5%) than without (3.5%) Covid-19 (*p* < 0.0001) and for those who died the median time to death was significantly longer for those with (14 days [8–21 days] Covid-19 than without it (4 days [1–15 days], *p* = 0.02).

### Comparison between those with Covid-19 diagnosed pre- and post-operatively

Of the patients with a post-operative diagnosis of Covid-19 (*n* = 35), 29 (82.9%) tested negative for Covid-19 at the time of surgery, where the Covid-19 status was not known or not tested in the remaining patients (likely due to being urgent/emergency cases). Similarly, of those with a pre-operative Covid-19 diagnosis (*n* = 17), all underwent urgent or emergency surgery.

Statistically significant differences are shown Table 2, highlighting that patients with a post-operative diagnosis of Covid-19 were older, had greater BMI, more likely to experience post-operative atrial fibrillation/flutter and had longer aortic cross clamp time than those diagnosed pre-operatively. They also were more likely to have experienced longer mechanical ventilation, and remain in hospital for 5 days longer for a total of 12 post-operative days than those diagnosed pre-operatively. Interestingly, there was no difference observed in ICU LOS or post-operative morbidity outcome. In-hospital mortality was 0% in those with pre-operatively diagnosed Covid-19, but 37.1% in those with a post-operative diagnosis (*p* < 0.0005). All other variables from Table 1 were explored and were statistically non-significant.

**Table 2:** Statistically significant differences between those diagnosed with Covid-19 pre-surgery compared to those diagnosed post-operatively. All definitions as per NACSA and ICNARC datasets

Variable	Covid-19 positive pre-surgery Frequency (%)/mean ± SD or Median[IQR] (*n* = 17)	Covid-19 positive post-surgery Frequency (%)/mean ± SD or Median[IQR] (*n* = 35)	p (Covid-19 positive vs Covid-19 negative)
**Demographics**			
Age (years/median [IQR])	60 [45–65]	70 [57–74]	0.029
Female gender	2 (11.8)	2 (5.7)	0.589
Ethnicity			
White	8 (80.0)	22 (95.7)	0.212
Asian	0 (0.0)	0 (0.0)	
Black	2 (20.0)	1 (4.4)	
Other	0 (0.0)	0 (0.0)	
**Cardiac risk factors**			
BMI (kg/m^2^/median [IQR])	25.6 [24.7–28.3]	27.4 [25–33]	0.084
**Examination and investigation**			
Pre-op heart rhythm			
Sinus rhythm	16 (94.1)	28 (80.0)	0.030
Atrial fibrillation/flutter	0 (0.0)	7 (20.0)	
Complete heart block/pacing	1 (5.9)	0 (0.0)	
Ventricular fibrillation/tachycardia	0 (0.0)	0 (0.0)	
Other abnormal rhythm	0 (0.0)	0 (0.0)	
**Pre-operative risk assessment**			
EuroSCORE (median [IQR])	2.8 [1.6–6.4]	4.9 [2.5–6.5]	0.400
**Intra-operative details**			
Aortic cross clamp time (minutes)	72 [45–84]	92 [75–139]	0.017
**Outcome**			
Number of days ventilated	1 [1–2]	2 [1–5]	0.017
Post-operative length of stay (days)	7 [5–11]	12 [7–21]	0.024
In-hospital mortality	0 (0)	13 (37.1)	0.005

#### Timing of deaths during the pandemic

**Fig. 2:**
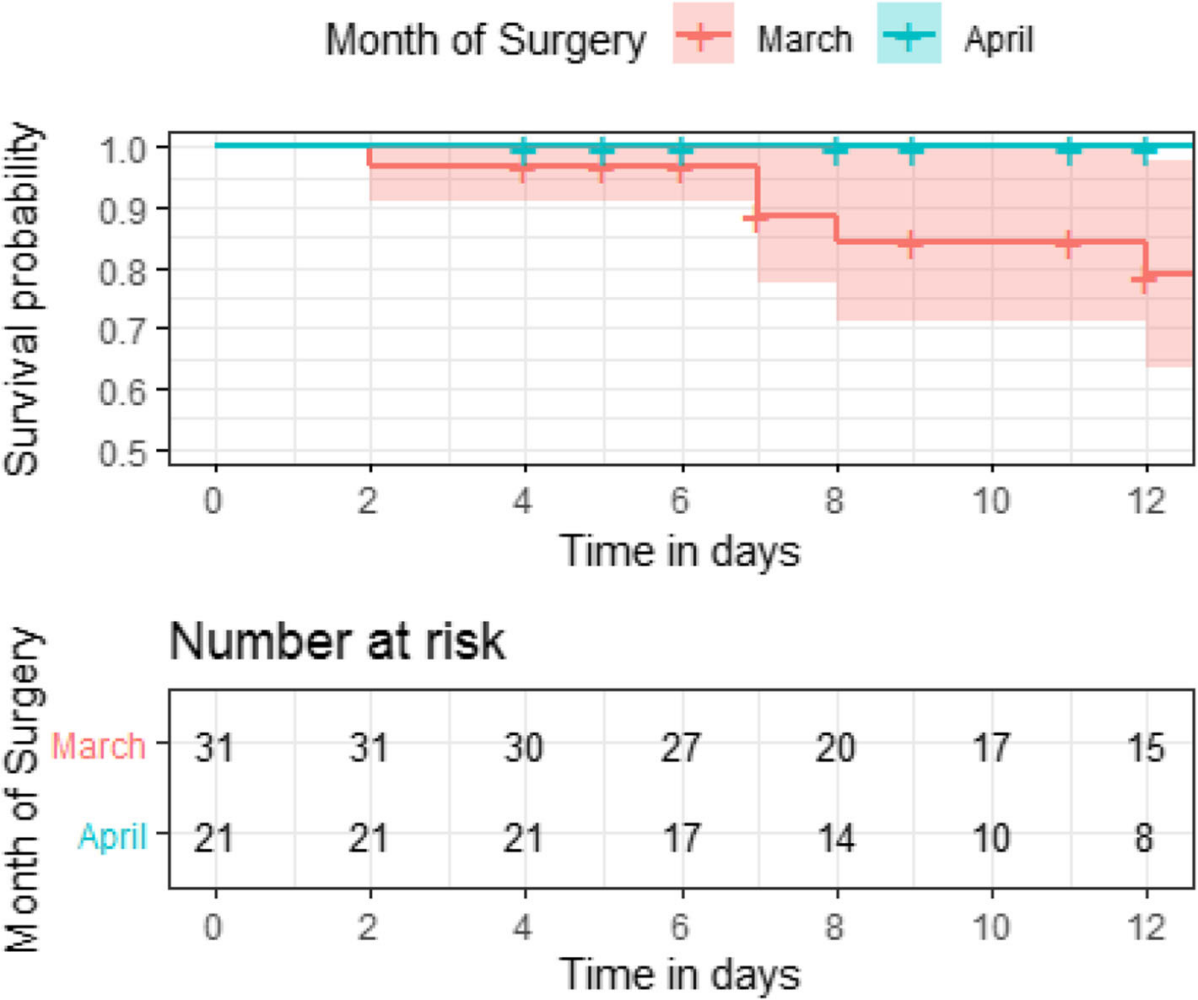
Survival from surgery in COVID-19 positive patients by month of surgery

Five patients died on the day of surgery (all Covid-19 negative), while 11 died in ICU (two were Covid-19 positive). As shown in Table 3 and Fig. 2, there is a trend suggesting that those with Covid-19 had greater chance of surviving surgery as time progresses, although this is not statistically significant.

**Table 3:** In-hospital mortality by month of surgery

Month of surgery	All (*n* = 755)	Covid-19 negative (*n* = 702)	Covid-19 positive (*n* = 53)
March 2020			
N(%)	27 (3.6)	17 (3.1)	10 (32.3)
Rate per 30 days (95%CI)	0.13 (0.09–0.20)	0.08 (0.05–0.14)	0.67 (0.36–1.25)
April 2020			
N(%)	9 (6.0)	6 (4.7)	3 (13.6)
Rate per 30 days (95%CI)	0.18 (0.08–0.37)	0.13 (0.05–0.33)	0.38 (0.12–1.16)
*P* value	*p* = 0.528	*p* = 0.107	*p* = 0.195

## Propensity score analysis for in-hospital mortality and post-operative length of stay

The propensity score matching confirms the significant difference in both in-hospital mortality and post-operative LOS between Covid-19 negative (*n* = 159) and positive patients (*n* = 53). In the propensity matched sets, 24.5% of those with Covid-19 died compared to 5.7% of those without Covid-19 (OR 5.65 (95%CI 2.21–15.04), *p* = 0.0005) while the difference in the median post-operative LOS between those with (11 days [7–15]) and without Covid-19 (8 days [6–13]) reduced from 5 days in the full dataset to 3 days after matching (95% CI 0.55–4.87, *p* = 0.019).

## Discussion

Our study, involving 755 patients undergoing cardiac surgery in nine centres during the early phase of the UK pandemic, has two key findings. Firstly, from propensity matched analysis we have shown that patients with Covid-19 had more than five times greater odds of dying than those without Covid-19, with a mortality rate of 24.5% v 3.5% (*p* < 0.0001). This is after accounting for factors found to be associated with Covid-19 (age [12], sex [13], ethnicity [13], diabetes [14], BMI [15]) and those with Covid-19 being more likely to undergo urgent and more complex surgery with associated longer intra-operative processes as a result. Secondly, those with a post-operative diagnosis of Covid-19 needed twice as much mechanical ventilation, remained in hospital for an additional 5 days and had a significantly increased risk of death compared to those with a pre-operative Covid-19 diagnosis (37.1% v 0.0% *p* = 0.005). Although those with a pre-operative diagnosis were younger and had a lower BMI, no differences were observed in the EuroSCORE and operative priority or type. Overall, pre-operatively diagnosed patients appeared to recover from surgery in a similar manner to non-Covid-19 patients and the burden of Covid-19 appears to particularly impact those diagnosed after surgery.

This pre- and post-surgery diagnosis divide has also been reported recently in a large global cohort (of which 7.6% underwent cardiac surgery), in both elective and emergency patients [10]. One theory is that surgery may accelerate progression of Covid-19 through an altered immune response [7], thus progressing the disease process in those pre-operatively incubating Covid-19. Equally, although the presence of pre-surgery Covid-19 may have led to the postponement of some surgery until negative Covid-19 swab results were obtained (especially for elective surgery patients), it is likely that if a patient was diagnosed pre-operatively, then operative processes, timing and treatment plans (for example, consultant-led procedures, reduction of blood products, avoiding/reducing CPB times) could be altered accordingly to reduce overall risk. However, our study was conducted at a time when little was known about Covid-19 and so these effects are likely to be minimal. Nevertheless, this will become much more important in the future now that treatment options are emerging, (for example, dexamethasone as reported in the RECOVERY Trial [16]) and more is known regarding the risk factors for Covid-19 and adverse outcomes.

It is entirely possible that the pre-and post-surgery diagnosis and adverse outcome divide is not a real phenomenon and simply reflects the poor and variable Covid-19 diagnosis procedures in the early phase of the UK pandemic. The swab false negative rate of up to 29% [17] could account for the large proportion of post-operative Covid-19 diagnoses in these early months. Certainly, although not significant, we have shown that mortality rate appears to decrease over time for Covid-19 positive patients. This is likely to be due to the revised pre-surgery swabbing protocols implemented in early April based on the early observed high mortality rate in patients with Covid-19 in March.

Our findings suggest that for future cardiac surgery there is a need for more robust and effective pre-hospitalisation and pre-surgery screening protocols to increase assurance of undergoing surgery Covid-free. Also, to further mitigate against the recovery and mortality burden for patients diagnosed post-operatively with Covid-19, Covid-free centres/hospitals with rigorous environment and staff screening protocols (for example, rigorous hygiene, social distancing and restricted access protocols) to ensure that status is maintained would be recommended. Dedicated cardiac surgery ‘hubs and spoke systems’ similar to that implemented in Italy [18], would be one such means of providing that. This is especially important as we identified a 52% reduction in cardiac surgery during this period, which is similar to that reported elsewhere [4]. This reduction of surgery, specifically the halting of elective surgery, will ultimately have a detrimental effect both on individuals’ waiting times (and their subsequent health deterioration) and health care organisations ability to manage the accumulation of patients, especially if subsequent Covid-19 peaks occur. This is not just a UK concern. Worldwide it is estimated that it could take between 29 and 95 weeks to rectify the over 28 million elective surgeries will have been estimated to have been cancelled during the 12-week pandemic peak, with a likely cancellation rate of around 81.7% for cardiac surgery [19]. Therefore, it is important that while we have to live with Covid-19 that patients are aware of the risks and that cardiac surgery services adapt, not only meet the demands of the pandemic, but to ensure the continuation of cardiac surgery as safely, but also as much, as possible.

### Strengths and limitations

This study has four main limitations. Firstly, not all UK centres participated in this study (reasons include lack of capacity to prepare and submit data) so our results may not be representative of the whole of the UK. Nevertheless we do have wide geographical representation covering England, Wales and Northern Ireland, including three large centres (Liverpool Heart and Chest Hospital, James Cook University Hospital and St Bartholomew’s Hospital). One of these (St Bartholomew’s Hospital) became the command centre for all cardiac surgery across London and parts of the South East of England [20], providing a similar ‘hub and spoke’ system referred to previously. Secondly, as previously eluded to, there were variable swabbing protocols across centres and Covid-19 diagnosis was initially reliant on a single negative swab pre-surgery. Due to the high false-negative rate this could account for the high incidence of post-operative Covid-19 diagnosis, particularly if surgery does indeed accelerate Covid-19 progression as suggested by Lei and colleagues [7]. Equally, to reflect clinical practice at the time we defined patients as covid-19 positive if no test were performed but was suspected clinically. Although only very few patients were included this way (one patient pre-operative diagnosis and five with a post-operative diagnosis), there is a potential that these could reflect other infectious complications. Nevertheless, as they have been recorded as covid-19 positive, they would have been clinically managed as such, and so we have also analysed them this way. Thirdly, our study reflects the initial snapshot of the outcomes of cardiac surgery during the Covid-19 pandemic, at a time when services, and the country, were adapting to the impact of Covid-19 in the UK and when personal protective equipment and staff and public Covid-19 tests were limited. Finally, as with any retrospective database analysis we are limited by the data collected within those existing datasets. Data pertaining to cause of death and also the timing of post-operative Covid-19 diagnosis, for example, would have been beneficial, but were not available at the time of analysis. To mitigate against some of these limitations, further work being undertaken focuses on the subsequent cardiac surgery response post April 2020, the longer-term implications and outcomes of having cardiac surgery during this time and whether the impact of a post-operative diagnosis remains following the revision of diagnosis processes and improvements in swab detection.

## Conclusions

Our study, thought to be the largest cohort study exploring cardiac surgery outcomes during a pandemic, identified that patients with Covid-19 had more than five times greater odds of dying than those without Covid-19. Those with a pre-operative diagnosis of Covid-19 appeared to recover from surgery in a similar manner to non-Covid-19 patients, but that the mortality burden of Covid-19 appears to particularly impact those diagnosed after surgery. To mitigate against these risks robust and effective pre-hospitalisation and pre-surgery diagnosis protocols are needed alongside effective strategies to ensure cardiac surgery environments remain Covid-19 free to reduce the post-operative risk. This could extend to implementing dedicated cardiac surgery hubs during future subsequent local or national Covid-19 peaks to ensure the safe and continual delivery of cardiac surgery.

## Data Availability

The datasets used and/or analysed during the current study are available from the corresponding author on reasonable request.

## Abbreviations

AWS: Amazon Web Service
BMI: Body Mass Index
CABG: Coronary Artery Bypass Graft
CCSC: Canadian Cardiovascular Society Classification
ICNARC: Intensive Care National Audit and Research Centre
ICU: Intensive Care Unit
IQR: Inter Quartile Range
LOS: Length of stay
LVEF: Left Ventricular Ejection Fraction
MI: Myocardial Infarction
NACSA: National Adult Cardiac Surgery Audit
NHS: National Health Service
NICOR: National Institute Cardiovascular Outcomes Research
NYHA: New York Heart Association classification
PCI: Percutaneous Coronary Intervention
SD: Standard Deviation
UK: United Kingdom
WHO: World Health Organisation
